# Women in Dentistry

**Published:** 1887-07

**Authors:** R. B. Ramsay

**Affiliations:** Pittsburg, Pa.


ARTICLE XI.

### WOMEN IN DENTISTRY.

MRS. R. B. RAMSAY, D. D. S., PITTSBURG, PA.

It would be interesting to study the various causes that led men and women, in the industrial world, to drift apart, till each were strangers to the other's work; and why, after the lapse of centuries, they are now uniting and working side by side.

Our imagination can date the dentist back to the primitive services of herbs and teas, hot salt, and hideous extractors, available to both sexes; but while the man advanced, secured the aid of science, established colleges to propagate learning, he pronounced his work a profession and constituted himself sole possessor and proprietor of the field; the woman continued the domestic treatment, propagated the superstitious arts, and submitted to his usurpation.

But some revolt occurred, and to-day women are given the freedom of dental colleges in America with the accompanying legal right to practice, and this privilege is mainly due to the efforts of a few noble men. Of these let me mention Professors Peirce and Truman, who, with opinions far in advance of their time, stemmed the tide of opposition (of no mean proportion, but of intelligent men whose convictions were to them strong proof of right) and with a Spartan courage persisted till they secured for women an inestimable privilege. Some day, when the full freedom of women shall be assured, the efforts of those who struggled to produce it will be a part of history.

Admitting women legally to practice is a check on the pernicious custom of the widows of dentists continuing the business, using the name and rights of the deceased, hiring incompetent men to transact the business.

Have women a right in dental schools? It is true these colleges were founded by men, and with money belonging to men, but some women bore these men, and some women helped earn this money; and since men have gone into domestic life for new avenues of labor, it is right that women should look to other fields for her life's work. But right or wrong, women dentists are an established fact; if every college door were closed against them, there are now women and money to start other schools.

But we do not anticipate a return to the old *regime*; for after a fair trial it is conceded that the presence of women in our colleges is beneficial to the young men, they show less disposition to rudeness, profanity and indulgence in evil habits which unfit them for professional gentlemen.

The profession generally has accepted the women gracefully, not as a necessary evil but as a power for good, knowing that the women who have joined their ranks are from a good social strata, and must eventually elevate the calling socially and professionally. Dental societies have given them a welcome and assigned them duties in public meetings. We should feel grateful to the profession for this privilege. At the door of Jefferson and the University of Pennsylvania she stands unnoticed, though holding her D. D. S. aloft and pointing to the arrangements made to admit this class on a third term, to receive the learning and benefits of M. D.

Are women an honor to the profession? Why should they not be? Those who have graduated have proved themselves apt pupils, entering the profession with an earnest endeavor to do her best. This is sure to bring esteem and success. Women are peculiarly adapted for the care, patience and delicate touch required in dental manipulations. The general public accepts the services of women. This is proven by the success of those following the business. That the women have not more fully availed themselves of the privileges is the result of long years of training called "womanly;" but this is a misnomer, for the world is ceasing to value a condition that in adversity means poverty and dependence. But women are awakening. In the Pennsylvania Dental College there are nine women enrolled this term as students. In the Philadelphia Dental College twenty-six have been graduated in the past, and in the present class there are seven enrolled and two or three others attending occasionally. The Baltimore College graduated three women and then refused further admission, nor will they to-day admit women as students. How manly!

Two of these thirty-two women graduates came from Germany, at a great expense, with attending sacrifices of leaving home and sojuurning alone in a strange country. They spent the time required by our colleges, and are welcomed home with greetings of honor and respect. They practice their art proud in the title of "American Dentist;" for thus they are styled by way of honorable mention, and a dentist there ranks above a physician in social caste.

The presence of women in the dental profession is one more star in the escutcheon of a profession that has advanced more rapidly from its birth than any other. Dentists have seemed to agree that nothing is impossible; prejudice has nowhere an abiding place. They are willing to cast out old ideas and accept new theories, put them to the test, and if good, assign them a place in the dental curriculum. Even so have they accepted women. They have extended to her the right hand of fellowship and given her a place in the front ranks.

Tell me the position of women in a nation and I will tell you the status history of that nation. The more intelligent and honored the women, the more highly civilized the nation. An ignorant and oppressed race of women are incapable of producing a race of civilized men. Women must be given her God-given privileges or the nation retrogrades. Show me the profession that turns a deaf ear to the knockings of this embodied spirit of civilization, and I will show you a calling whose precepts and practices are but a shade from the dark ages. This 19th century, more than any in the past, is one of change—constant, persistent change. Everything in science, religion and art, is being tested. The next century will dawn on a glorious state of unity, and surely the women will be there.—*Items of Interest.*

---

### THE AMERICAN DENTAL ASSOCIATION.

